# Impact of Hot-Melt Extrusion on Glibenclamide’s Physical and Chemical States and Dissolution Behavior: Case Studies with Three Polymer Blend Matrices

**DOI:** 10.3390/pharmaceutics16081071

**Published:** 2024-08-15

**Authors:** Nina Zupan, Ines Yous, Florence Danede, Jeremy Verin, Mostafa Kouach, Catherine Foulon, Emeline Dudognon, Susanne Florin Muschert

**Affiliations:** 1 Univ. Lille, Inserm, CHU Lille, U1008, F-59000 Lille, France; nina.zupan@univ-lille.fr (N.Z.);; 2 Univ. Lille, CNRS, INRAE, Centrale Lille, UMR 8207-UMET, F-59000 Lille, Franceemeline.dudognon@univ-lille.fr (E.D.); 3 Univ. Lille, CHU Lille, ULR 7365-GRITA, F-59000 Lille, France

**Keywords:** hot-melt extrusion, solid dispersion, ternary blends, degradation, glibenclamide, sustained drug release

## Abstract

This research work dives into the complexity of hot-melt extrusion (HME) and its influence on drug stability, focusing on solid dispersions containing 30% of glibenclamide and three 50:50 polymer blends. The polymers used in the study are Ethocel Standard 10 Premium, Kollidon SR and Affinisol HPMC HME 4M. Glibenclamide solid dispersions are characterized using thermal analyses (thermogravimetric analysis (TGA) and differential scanning calorimetry), X-ray diffraction and scanning electron microscopy. This study reveals the transformation of glibenclamide into impurity A during the HME process using mass spectrometry and TGA. Thus, it enables the quantification of the extent of degradation. Furthermore, this work shows how polymer–polymer blend matrices exert an impact on process parameters, the active pharmaceutical ingredient’s physical state, and drug release behavior. In vitro dissolution studies show that the polymeric matrices investigated provide extended drug release (over 24 h), mainly dictated by the polymer’s chemical nature. This paper highlights how glibenclamide is degraded during HME and how polymer selection crucially affects the sustained release dynamics.

## 1. Introduction

Polymers are widely used to formulate oral solid dosage forms with modified drug release. Their physicochemical properties are crucial for assessing their ability to sustain drug release. An appropriate combination of polymers can create matrices that provide the desired sustained release properties and enhance the physical stability of the drug within the polymer–polymer blend matrix [1,2]. The hot-melt extrusion (HME) technique has become a recognized, continuous, solvent-free process in the product development of pharmaceutical dosage forms and innovative new dosage forms, where the active pharmaceutical ingredient (API) is dispersed into polymer (blend) matrices [3,4,5,6]. During the HME process, the API can either remain in a crystalline state or transform into the amorphous form, which shows better bioavailability. However, the amorphous state is thermodynamically unstable and challenging to maintain. Therefore, polymers also play a crucial role as carriers to prevent API recrystallization and stabilize the amorphous form in polymer matrices [7,8]. Besides the possibility of enhancing the drug’s oral bioavailability, HME has been demonstrated as a viable process for the preparation of sustained-release tablets, pellets, granules and injection molding [9,10]. Additionally, the HME process has been used in recent years to produce drug-loaded filaments via HME, enabling three-dimensional printing to create pharmaceutical forms with customized properties such as shape, size, dosage, and release kinetics. This advancement represents a step toward personalized drug therapies for individual patients [11,12]. However, the components of hot-melt extruded formulations must possess thermoplastic properties and exhibit thermal stability [2,13,14].

Indeed, the hot-melt extrusion process represents a challenge to process high-shear energy-dependent or heat-labile drugs [15]. The transformation of the physical state of the drug relies on mechanical and thermal energy inputs [16]. The energy needed for the transformation from the crystalline to amorphous state can cause a degradation of heat-labile drugs. Moreover, poorly stable drugs are susceptible to different degradation processes, including dehydration, isomerization, and, most commonly, hydrolysis and oxidation [9,17]. Chemical degradation can be generated within the HME process by the presence of moisture, oxygen and elevated temperatures [15,18]. Thermally induced degradation is provided by the external heat applied to the barrel [19]. In addition, mechanical energy is induced through the interplay of the screw elements and material within the barrel, which can also be converted to thermal energy. Thus, it becomes challenging to control the energy generated during the process [20]. Our model drug glibenclamide (GLB), also known as glyburide, is an antidiabetic drug rated as a class II compound of the Biopharmaceutics Classification System (BCS) due to its very poor water solubility and high permeability [21,22]. It is a crystalline compound with a melting temperature equal to Tm = 174 °C [23] that can also be amorphized by the classical quench cooling of the melt [22,24,25] or by milling [22,25]. However, according to Patterson et al. [25], with the first amorphization route, heating GLB above its melting point induces thermal degradation. This indicates that GLB is a heat-labile drug that contains two molecular parts susceptible to undergoing thermal degradation under severe conditions: on the one hand, the sulfonylurea part and, on the other hand, the benzamide group [26].

The main objective of the present study was to reach a better understanding of the impact of the hot-melt extrusion technique on the physical state, the chemical changes and the dissolution behavior of glibenclamide when extruded with different polymer blends at a temperature below the melting temperature of GLB. Firstly, the degradation of GLB during the HME process was assessed. Then, the physical state of the processed blends was characterized, and finally, the dissolution behavior of GLB within the different matrices was investigated in a two-stage dissolution set-up with in vitro dissolution media of varying pH.

## 2. Materials and Methods

### 2.1. Materials

Glibenclamide (CAS number: 10238-21-8) was purchased from Sri Krishna Pharma (99.9% pure, Hyderabad, India). Impurity A of GLB (CAS number: 21165-77-5) was purchased from Merck (99.9% pure, Strasbourg, France). The materials were used as received. Three different polymers were used: ethyl cellulose (EC) under the name Ethocel Standard 10 Premium and hydroxy-propyl methyl cellulose (HPMC) under the trade name Affinisol HPMC HME 4M were kind gifts from Colorcon (Dartford, UK); polyvinyl pyrrolidone/polyvinyl acetate (PVP/PVPAc) branded as Kollidon SR was donated from BASF (Ludwigshafen, Germany). Kollidon SR is composed of 20 wt.% PVP and 80 wt.% PVAc. Phosphoric acid and sodium hydroxide were purchased from Sigma Aldrich (Seelze, Germany). Hydrochloric acid (32%) and monobasic potassium phosphate from Fisher Scientific, UK. Ultra-pure water was obtained from Veolia (Vendin le Vieil, France) and acetonitrile (HPLC grade) from Carlo Erba (Val de Reuil, France).

### 2.2. Hot-Melt Extrusion

A Pharma 11 (Thermo Fisher Scientific, Karlsruhe, Germany) equipped with co-rotating twin screws (11 mm diameter) and a heated die of 2 mm diameter was used. A total of 30 wt.% of glibenclamide (GLB) was blended with 35 wt.% of one polymer and 35 wt.% of another polymer. Three ternary blends were thus formulated: GLB and HPMC and PVP/PVAc, GLB and EC and PVP/PVAc, and GLB and HPMC and EC. The blends were pre-mixed with a powder shaker mixer, type Turbula T2C, for 5 min at a speed of 42 rpm. The process temperatures and screw speeds were adapted for each blend. The screw configuration (Figure S1 in the Supplementary Materials) was maintained constant, featuring two distinct zones of mixing elements. Both mixing sections were formed by combining initial mixing elements at an offset angle of 30°, followed by elements at 60° and 90°. The first mixing zone consisted of three elements at 30°, five at 60°, and four elements at 90°. The second mixing zone comprised three elements at 30°, three at 60°, and four at 90°.

Gravitational feeding was performed manually. The feeding rate was determined by dividing the weight of the powder blend fed over a specified time period (approximately 10 min). After cooling, the hot melt extrudates were analyzed.

Extrudate output was measured by conducting the extrusion process for approximately 10 min after the process stabilization. The collected extrudate was weighed, and the obtained value was divided by the collection time to determine the output rate.

Specific mechanical energy (*SME*) was calculated from the processing parameters using the following Equation (1).

(1) $$SME=\frac{P_{m}}{ṁ}=\frac{2\cdot \pi \cdot n\cdot M}{ṁ},$$

where *P_m_* is the mechanical power at the feeding rate *ṁ* (kg/h). The mechanical power can also be further developed with screw speed (*n*) expressed in rpm and torque (*M*) in Nm [27].

### 2.3. Thermogravimetric Analysis (TGA)

TGA measurements were performed using the Q500 apparatus from TA instruments (Guyancourt, France). A small sample (between 5 and 8 mg) was placed into an open aluminum pan on a weighing scale. Both the sample and the weighing scale were kept in a dry atmosphere using a constant flow rate (50 mL/min) of highly pure nitrogen gas (99.999%). The temperature reading was calibrated with the measurements of the Curie points of alumel and nickel (provided by TA Instruments). The mass reading was calibrated using certified calibration weights (TA Instruments). All scans were performed at 5 °C/min from room temperature to 420 °C.

### 2.4. Temperature Modulated Differential Scanning Calorimetry (MDSC)

Temperature-modulated differential scanning calorimetry (MDSC) was performed with a DSC Q20 (TA Instruments) connected to a refrigerated cooling system. Open aluminum pans were used to contain the small amounts of samples (approximately 3–5 mg) to provide good thermal conductivity and sharp enthalpic events. The measurement cell was purged with dry nitrogen gas (99.999% pure) at a flow rate of 50 mL/min during the analysis. Temperatures and enthalpies were calibrated using indium at the same heating rate and under the same environmental conditions as the experiments. Specific heat capacity was measured using sapphire as a reference by applying the same heating rate and modulated parameters as for the experiments: all scans were performed using an average heating rate of 5 °C/min with a temperature modulation of ±0.531 °C per 40 s in the range between 10 °C and 170 °C.

### 2.5. X-ray Diffraction (XRD)

The XRD experiments were performed with an XPERT PRO MPD diffractometer in Debye−Scherrer geometry (λ_Cu Kα_ = 1.5406 Å) equipped with an X’celerator linear position-sensitive detector. For analyses, samples (powder or pieces of extrudates) were placed on a plate. X-ray diagrams were recorded from 6 to 46° (2θ) at room temperature, with a scan step of 0.0167°/s.

### 2.6. High-Performance Liquid Chromatography (HPLC) Analysis and Drug Content Determination

The drug content determination was performed using HPLC-UV analysis using a Thermo Fisher Scientific Ultimate 3000 Series HPLC, equipped with an LPG 3400 SD/RS pump, an auto sampler (WPS-3000 SL) and a UV–Vis detector (VWD-3400RS) (Thermo Fisher Scientific, Waltham, MA, USA). A reversed-phase column C18 (Gemini 5 μm; 110 Å; 150 × 4.6 mm; Phenomenex, Le Pecq, France) was used. An isocratic method consisting of 55% A (38.3 mM solution with pH 3.5 made from phosphoric acid (purity > 98.0%) and 0.5% triethylamine) and 45% B (100% acetonitrile, HPLC grade) was developed. The sampler compartment was kept at 25 °C, and the column was heated to 40 °C. The detection wavelength was set to 228 nm, and the flow rate to 1.5 mL/min. This method was developed in-house. Samples were filtered using PTFE membrane filters 0.45 μm (Agilent Technologies, Qingdao, China). Twenty microliters of samples were injected. Calibration graphs were plotted for the drug, and linearity was demonstrated from 0.1 to 25 μg/L (R^2^ ≥ 0.999) concentration ranges. All time points have been above the LOD (limit of detection). The LOQ (limit of quantification) was determined at a signal-to-noise ratio of 15:1 being 0.2 µg/mL, and the LOD was determined to be 0.1 µg/mL at a signal-to-noise ratio of 5:1.

Extrudates were dissolved in 40 mL ethanol (99.9% pure) and left overnight at 37 °C in a shaker set to 80 rpm. Ethanolic solutions of extrudates were filtered using PTFE membrane filters 0.45 μm (Agilent Technologies). The drug content was determined through the analysis of the peak appearing at a run time of 9.9 min, while degraded GLB (impurity A) concentrations were determined from the analysis of the peak appearing at 2.9 min. All experiments were conducted in triplicate: the first sample was taken from the early parts of the filament, the second from the middle, and the final sample from the end of the obtained extrudate to assess the drug content homogeneity within the extrudates. Mean values ± standard deviations are reported.

Drug content recovery (%) was calculated using the following Equation (2):

(2) $$Drug\;recovery\;\left(\%\right)=\frac{amount\;of\;drug\;recovered}{initial\;amount\;of\;drug}\times 100,$$

where the *initial amount of drug* equals the initial mass of GLB, which represents 30% of the total extrudate weight, and the *amount of drug recovered* was obtained from the concentration determined by the HPLC analysis.

### 2.7. Mass Spectrometry (MS) Analysis

High-resolution MS analysis was performed in full scan negative mode using a Thermo Scientific Orbitrap Mass Spectrometer Exactive equipped with a heated electrospray ionization source (HESI-II). Mass spectra were recorded at a high resolution of 100,000. Instrument control, data acquisition and processing were performed using the associated Xcalibur 2.2 and Exactive 1.1 software.

MS and tandem mass spectrometry (MS-MS) analyses were carried out with a QTRAP 5500 MS/MS hybrid system triple quadrupole/linear ion trap mass spectrometer (AB Sciex, Foster City, CA, USA) equipped with a Turbo VTM ion source. Instrument control, data acquisition and processing were performed using Analyst 1.7.2 software. MS analysis was carried out in negative ionization mode (ion spray voltage, −4500 V).

### 2.8. Determination of the Saturation Concentration and the Two-Stage Glibenclamide Dissolution Kinetics

The evolution of the saturation concentration of the API was observed over a total period of 168 h in order to quantify its solubility limit. For that purpose, the traditional shake flask method was used [28]. Excess amounts of API in powder form (about 100 mg) were exposed to 30 mL of the investigated media: HCl solution (pH 1.2) and phosphate buffer (PB, pH 6.8) under the addition of 1% of surfactant Polysorbate 80 in amber flasks. These flasks were placed into a horizontal shaker (37 °C, 80 rpm; GFL 3033; Gesellschaft fuer Labortechnik, Burgwedel, Germany). At predetermined time points, 600 μL samples were withdrawn through a 5 μm syringe filter (BD Blunt Fill Needle with Filter, 18 G ×11/2; Franklin Lakes, NJ, USA) and subsequently filtered through a 0.45 μm PTFE membrane filter (Agilent Technologies) into a 1.5 mL Eppendorf tubes. The samples were then diluted 10 times directly into vials for HPLC analysis. All experiments were conducted in triplicate. Mean values ± standard deviations are reported. The saturation concentration values began reaching a plateau at around 24 h; here, the 48 h values are reported.

Dissolution kinetics studies were performed on cylindrical extrudate samples obtained from HME, containing 30 wt.% glibenclamide, in a USP apparatus II (AT7 Smart, Sotax, Basel, Switzerland) with 6 vessels and 500 mL of “standard” in vitro dissolution medium per vessel. The paddle speed was set to 75 rpm, and the temperature of the medium was maintained at 37 ± 0.5 °C. Two-milliliter samples were withdrawn manually and filtered through a 5 μm syringe filter (BD Blunt Fill Needle with Filter, 18 G ×11/2) at predetermined time points (after 10, 20, 30, 45 min and then after 1, 1.5, 2, 2.5, 3, 3.5, 4, 5, 6, 7, 8 and 24 h). The samples were filtered again through a 0.45 μm PTFE membrane filter (Agilent Technologies), and the first milliliter was returned to the respective dissolution vessel, while the remaining 1 mL was transferred into an Eppendorf tube and immediately diluted with mobile phase to prevent precipitation prior to HPLC analysis. Dissolution tests were performed in 500 mL of HCl solution (pH 1.2) for the first 30 min, and the solution was subsequently replaced with 500 mL of phosphate buffer (pH 6.8) that imitates the pH of intestinal fluid. Polysorbate 80 was added as a surfactant to all the media to increase the release and detectability of glibenclamide in HPLC. All experiments were conducted in triplicate. Mean values ± standard deviations are reported.

### 2.9. Water Content and Dry Mass Loss Kinetics of the Extrudates

The water content and the dry mass loss kinetics of the extrudates were determined upon exposure to phosphate buffer (pH 6.8), simulating the pH of the one that can be found in intestinal fluids. Extrudates were placed into flasks (one sample per flask), filled with 50 mL of phosphate buffer pH 6.8 and agitated at 80 rpm in a horizontal shaker at 37 °C (GFL 3033). At predetermined time points, extrudate samples were withdrawn from the phosphate buffer, and the excess media was removed by careful blotting with Kimtech Precision Wipes (Kimberly-Clark, Irving, TX, USA). The extrudates were accurately weighed (wet mass) and dried to constant weight at 60 °C (dry mass).

The water uptake and the dry mass (%) at time *t* were calculated as follows:

(3) $$Water\;content\;\left(\%\right)\left(t\right)=\frac{wet\;mass\;\left(t\right)-dry\;mass\;\left(t\right)}{wet\;mass\;\left(t\right)}\times 100\%,$$

(4) $$Dry\;mass\;\left(\%\right)\left(t\right)=\frac{dry\;mass\;\left(t\right)}{dry\;mass\;\left(t=0\right)}\times 100\%,$$

where *dry mass* (*t* = 0) is the mass of the dry extrudate before exposure to the medium. All experiments were performed in triplicate. Mean values ± standard deviations are reported.

### 2.10. Optical Imaging of Extrudates

Pictures of the extrudates before and after dissolution studies (samples dried at 20 °C in a desiccator filled with silica gel granules) were taken with an SZN-6 trinocular stereo zoom microscope (Optika, Ponteranica, Italy) equipped with an optical camera (Optika Vision Lite 2.1 software). Reflection mode was used as the light source. The lengths and diameters of the extrudates were determined using AxioVision 4.8.2 software (Carl Zeiss Microscopy, Oberkochen, Germany).

### 2.11. Scanning Electron Microscopy (SEM) Coupled with Energy Dispersive X-ray (EDX)

SEM pictures were taken to determine the morphology of the particles using a JEOL Field Emission apparatus (JSM-7800F, Tokyo, Japan). Samples were mounted on an SEM stub using double-sided adhesive carbon ribbon. To avoid surface charging, samples were chromium-coated (100 Å) by electro-sputtering under vacuum prior to SEM observations. The observations were performed at a 6 kV acceleration voltage, with a small probe current and the lowest objective lens aperture to reduce beam damage on the sample surface. SEM imaging was optionally coupled with EDX microanalysis (XMax SDD detector, Aztec 6.1 software, Oxford Instruments, Oxon, UK).

## 3. Results and Discussion

### 3.1. Hot-Melt Extrusion Process Parameters

The three ternary blends GLB and HPMC and PVP/PVAc, GLB and EC and PVP/PVAc, and GLB and HPMC and EC were prepared as described in Section 2.2. These three different formulations were introduced into the extruder with a screw configuration, including two mixing zones, to ensure sufficient blending and introduction of shear energy (see Supplementary Materials, Figure S1). Both mixing zones are composed of the same kneading elements, which had offset angles of 30°, 60° and 90°. However, different process conditions were used depending on the composition of the polymeric matrix within which the API is embedded. One criterion to validate the processing parameters of the extrusion was to limit the torque: the conditions (screw speed, temperature and feeding rate) were adapted to keep the torque between 30% and 80%. Moreover, the temperature in the barrel had to be maintained below the melting point of glibenclamide (Tm = 174 °C) since it was reported by Patterson et al. [25] that the drug undergoes significant chemical degradation when exceeding the melting temperature. The visual appearance of the extrudate at the exit was also an important criterion for obtaining smooth and straight extrudates without the presence of shark skinning. Regarding the feeding rate, it was controlled to avoid any material build-up in the feeding zone. Processing parameters and measurements are presented in Table 1.

All three formulations were extruded below the melting point of glibenclamide as a first attempt to prevent degradation, with the highest temperature applied being 155 °C at the die. When comparing the three formulations, GLB and EC and PVP/PVAc was extruded at the highest screw speed (200 rpm) and feeding rate (1.92 g/min), resulting in the lowest torque (40%) and an extrudate output of 1.85 g/min. The blend GLB and HPMC and EC were performed at the lowest feeding rate (1.06 g/min) at a screw speed of 45 rpm, resulting in the lowest extrudate output (0.81 g/min) and highest torque (75%); a further increase in the feeding rate would have blocked the process.

The blend GLB and HPMC and PVP/PVAc was extruded with a feeding rate of 1.71 g/min at 160 rpm, resulting in an extrudate output higher than that of the formulation GLB and HPMC and EC at a relatively low temperature ranging between 110 and 115 °C in the sheath (increasing up to 130 °C at the die for a smoother extrudate). As reported by Gupta et al., the shear rate has a major impact on the viscosity of the HPMC grade used in this study. The viscosity significantly decreases with higher shear, meaning it exhibits a shear-thinning behavior [29]. This suggests that the higher screw speed used in the blend GLB and HPMC and PVP/PVAc compared with the one used for GLB and HPMC and EC increases the shear and reduces the melt viscosity of the HPMC, consequently resulting in lower torque.

**Figure 1:**
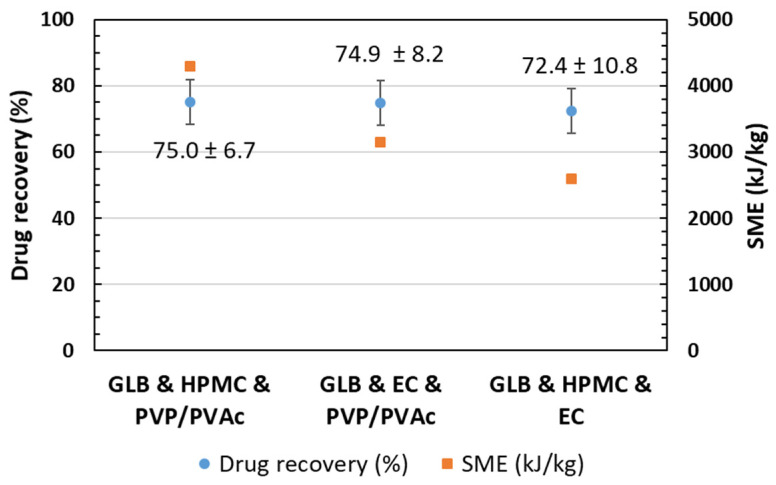
Glibenclamide drug recovery in the extrudates and the specific mechanical energy input during the runs of the three different formulations.

Moreover, Huang et al. reported that a faster feeding rate could help increase drug recovery due to a shorter residence time and, hence, less shear and heat exposure [30]. It is important to limit the temperature to avoid thermal degradation. Nevertheless, setting the process to low temperatures can result in high energy and greater shear stress due to the higher viscosity of the polymer [31]. In order to investigate if GLB was exposed to the degradation process during the extrusion, the drug recovery analyses were performed using the UV-HPLC technique. By UV-HPLC (cf. 2.7), the signature of GLB appeared as a peak after 9.9 min of elution. However, for all three formulations, in addition to this peak, another peak was observed at 2.9 min, indicating degradation of GLB. To determine the drug recovery, the drug content of the extrudate was quantified from the analysis (cf. 2.7.) of the peak at the run time of 9.9 min, identified as non-degraded GLB, and divided by the initial content (30 wt.%). As shown in Table 1 and Figure 1, a recovery lower than 100% was obtained. However, the drug recovery was approximately the same for all three formulations, around 75%. The degradation or loss of product can be attributed to factors such as temperature and high shear stress. As already reported by Martin [32], the real temperature that the material experiences inside the extruder chamber can differ from the temperatures set and can be much higher than presented in Table 1. Substantial heat can be induced inside the barrel by the shear stress and pressure, especially at the kneading elements. This induces energy, which increases the temperature of the melt, which cannot be detected by the thermal detectors successfully since they are located within the barrel wall and do not measure the real material temperature directly on the screw [32]. The highest shear stress and consequently higher temperatures occur in the areas of kneading elements (with offset angles of 30°, 60° and 90°). The lower drug recovery can be assigned to these induced localized higher temperatures.

In addition, specific mechanical energy was investigated (cf. 2.2). It is defined as the amount of mechanical energy applied using the extruder motor to a system per unit mass of material processed [33]. As a result, this parameter could assist in determining the limit of energy applied to avoid degradation [34]. SME values ranged approximately from 2600 to 4300 kJ/kg (cf. Figure 1). The blend GLB and EC and HPMC was extruded at 155 °C resulting in an SME value of 2600 kJ/kg, while the blends of GLB with EC and PVP/PVAc and HPMC and PVP/PVAc were extruded at lower temperatures (130 °C) and resulted in higher SME values (>3200 kJ/kg). Surprisingly, despite this difference in SME values, the drug recovery results in similar values (approx. 75%) for all three formulations. It was anticipated that the drug recovery would increase as the SME decreased; however, this study highlights that this is not the case. The resulting SME appears to be too high to avoid degradation in all three formulations.

The chemical analysis of the extrudates has shown that GLB undergoes degradation during the HME process for the three formulations investigated. The results show that the polymer matrix plays a role in process parameters and, thus, in the SME, although the amount of “non-degraded” drug (recovery) remains the same. However, comparing the properties of the products induced by extrusion for the three formulations is challenging, as the nature of the different polymeric matrices, in addition to a variation in process parameters, can also induce different interactions with the API and modify the physical states. 

### 3.2. The Thermal Degradation of Glibenclamide

**Figure 2:**
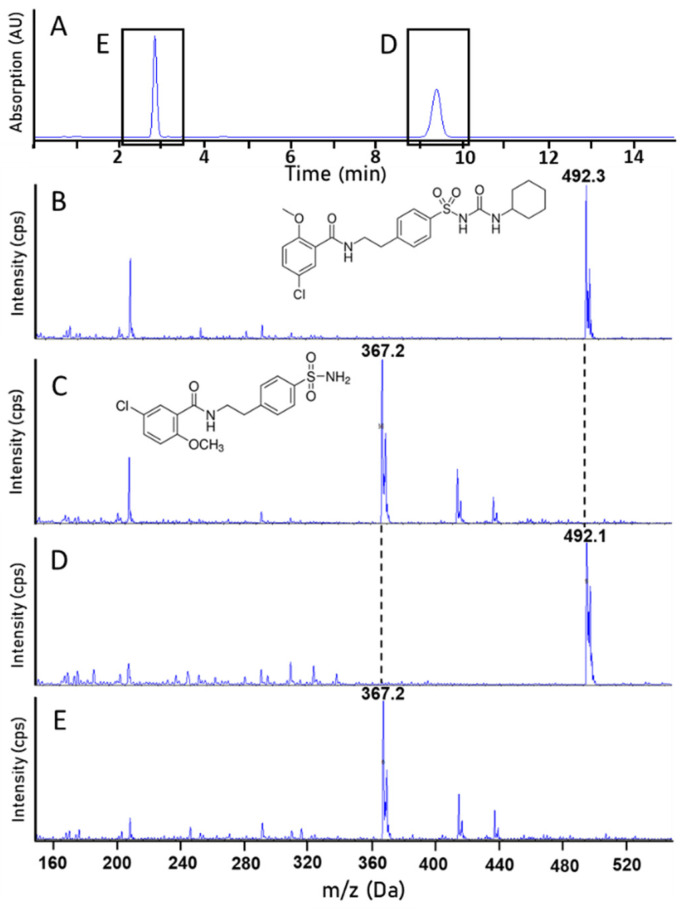
Analyses of mass spectra to identify components present in the extrudate formulation GLB and HPMC and PVP/PVAc obtained under process conditions defined in Table 1. (**A**) represents the UV-HPLC chromatogram of the extruded formulation. Further, the mass spectra of (**B**) glibenclamide (as received), (**C**) impurity A (as received), (**D**) the compound eluted at t = 9.9 min (from ethanolic solution of the extrudate), and (**E**) the compound eluted at t = 2.9 min (from ethanolic solution of the extrudate) are shown.

As mentioned in the previous section, the HPLC analysis of extrudate composed of GLB, HPMC and PVP/PVAc showed two peaks appearing at t = 2.9 min and 9.9 min (see Figure 2A), indicating a degradation of GLB during the HME process. In order to identify the degradation products, an MS-MS analysis of the compounds eluted at times 9.9 min and 2.9 min was performed in negative mode and is presented, respectively, in Figure 2D,E. This allowed the identification of the degradation product of glibenclamide after being processed with HME. Indeed, the results revealed the presence of two different products: GLB and a related substance. First, the signal observed in Figure 2D, at *m*/*z* 492 Da, corresponds to the GLB standard reference (Figure 2B), which indicates the presence of intact glibenclamide after the HME process. Secondly, the mass spectra of the HME product eluted at 2.9 min showed a signal corresponding to an ion of *m*/*z* 367.2 Da (Figure 2E). HPLC analysis of glibenclamide impurity A (reference standard) with a molecular weight of 368.84 g/mol was performed under the same experimental conditions. A chromatographic peak was detected at 2.9 min. The mass spectra of this compound (Figure 2C) were equivalent to the spectra observed for the HME product. This indicates that impurity A was formed during the thermal degradation of glibenclamide in the HME process. Systematic forced degradation of glibenclamide has already been performed and reported under various conditions. The most common degradation pathway of glibenclamide proposed is the hydrolysis of sulfonylureas [17,35]. Here, the small amount of water present in hygroscopic polymers could attack the carbonyl carbon, which would result in hydrolyzing the glibenclamide to a sulfonamide (impurity A). Huang et al. reported a similar effect of hydrolysis during HME with another antidiabetic drug (gliclazide) and HPMC in the presence of 1.1% water [15]. It should be noted that unprocessed glibenclamide and the degradation product impurity A both exhibit the same UV response at 228 nm. Therefore, liquid chromatography is necessary to determine the drug recovery from the samples obtained with the HME process since UV/VIS spectrometry cannot distinguish between both products.

### 3.3. Physical State Analysis

**Figure 3:**
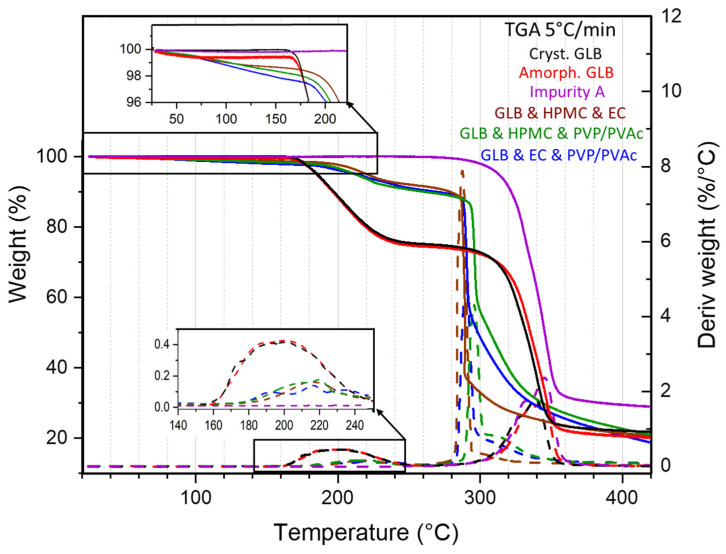
Thermogravimetric analyses (weight evolution and its temperature first derivative) performed at 5 °C/min of extrudates made from ternary blends containing 30% GLB. Thermograms of crystalline and amorphous GLB powder and purchased impurity A were added for comparison. The dashed curves represent the temperature derivative of the signal.

Thermogravimetric analyses were conducted upon heating at 5 °C/min on glibenclamide (both the crystalline and the amorphous form), impurity A and the extruded ternary blend formulations. TGA profiles are reported in Figure 3. For glibenclamide, the temperature derivative of the signal shows that the weight loss, usually associated with thermal degradation, begins at 160 °C regardless of the physical state. This is consistent with observations made by Patterson et al. [25] reporting chemical degradation (of up to 16%) of a GLB sample heated above 174 °C, which was identified to be the melting temperature. In Figure 3, it can be seen that the weight loss proceeds in two stages: from 160 °C to 240 °C (~25%) and from 280 °C to 360 °C. Comparison with the TGA profile of impurity A shows that the second stage corresponds to the thermal degradation of impurity A. This allows us to conclude that at 280 °C, all GLB molecules are degraded, leaving only impurity A molecules. Thus, the first stage of weight loss corresponds to the degradation of the other part of GLB molecules: the cyclohexyl isocyanate moiety. This is corroborated by the magnitude of this weight loss (25%), which matches the ratio of the molecular weights of cyclohexyl isocyanate and GLB. Additionally, the thermograms show that, while the crystalline form of GLB exhibited no weight loss below 160 °C, a slight weight loss of 0.4% was observed up to 100 °C for the amorphous GLB obtained by milling at room temperature. This is attributable to the loss of free water adsorbed on the sample’s surface, which is commonly observed in milled compounds due to their higher surface reactivity.

The TGA profiles of the extruded blends showed several stages of weight loss. Firstly, a continuous and small weight loss was observed between room temperature and 150 °C due to water loss. Compared with amorphous GLB, this water loss was more significant and continued until higher temperatures, indicating the evaporation of water adsorbed on the surface of the extrudate up to 100 °C (~1.2%), followed by the departure of water entrapped within the hygroscopic polymeric matrices that remained despite heating during the HME process. At 150 °C, the weight loss reaches almost 2%.

However, Figure 3 shows that the weight loss occurred at higher temperatures and proceeded in two stages: from 180 °C to 280 °C and from 280 °C to 360 °C. The weight loss between 180 °C and 280 °C corresponds to the first step of the weight loss of GLB, i.e., the degradation of the cyclohexyl isocyanate moiety, since the temperature range and the magnitude agree (the 7% weight loss roughly corresponds to the quarter of the GLB critically constrained in the formulations). The magnitude of this loss remained unaffected by the reduced fraction of GLB remaining in the formulations after the HME process. This suggests that during HME, some GLB molecules dissociated into impurity A and cyclohexyl isocyanate, but these degradation products stayed within the formulations. It should be underlined that TGA, by measuring weight loss, enables the detection of vaporization of degradation products, which may not necessarily correspond to the formation of these products. The vaporization temperature of cyclohexyl isocyanate is reported to be 170 °C [36]; thus, the decomposition of GLB into impurity A and cyclohexyl isocyanate cannot be detected by TGA if it occurs at lower temperatures, such as the extrusion temperatures applied in this study. It can be noted that since the vaporization temperature of cyclohexylamine is reported to be 135 °C [37], the results indicate that this compound was not formed during HME. At higher temperatures, the second stage of weight loss corresponds to the ongoing degradation of GLB (impurity A) and the degradation of the polymeric matrices. This step occurred mainly between 280 °C and 300 °C, which corresponds to lower temperatures compared with the pure compounds (above 300 °C). This can be explained by reactions between the degradation products of GLB and the polymeric matrices that could accelerate the thermal degradation of the blends.

**Figure 4:**
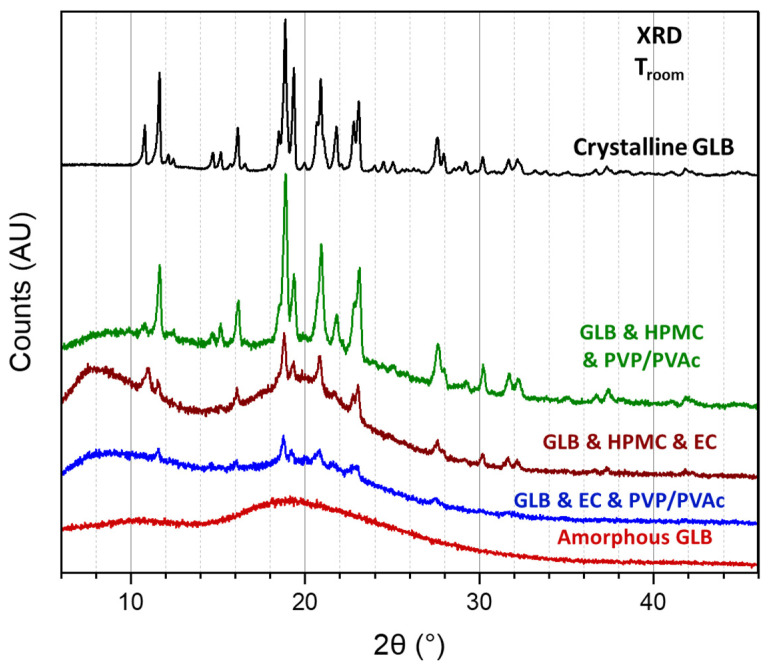
X-ray diffraction patterns recorded at room temperature of extrudates made from ternary blends containing 30% GLB. Diffractograms of crystalline and amorphous GLB are added for comparison.

Furthermore, the three investigated ternary blends were analyzed using X-ray diffraction (XRD) to determine the physical state of our model drug within the polymeric matrices after the process of extrusion. The black curve in Figure 4 shows the XRD patterns of commercial glibenclamide (used as received) recorded at room temperature. The recorded scan shows Bragg peaks, characteristic of the crystalline structure, which was already resolved by Suresh and co-workers [38]. For comparison, in the pattern of glassy GLB (obtained by milling at room temperature) represented at the bottom of Figure 4, only a diffusion halo can be seen. This is characteristic of the amorphous form since there are no more constructive/destructive interferences of the scattered X-ray waves due to the periodicity of the crystal lattice. The XRD patterns of the ternary blends show the diffusion halo expected for polymeric matrices, as well as Bragg peaks corresponding to the GLB crystalline form, indicating the presence of remaining crystalline glibenclamide. However, in the case of GLB and EC and PVP/PVAc and GLB and HPMC and EC formulations, the low peak intensities indicate a reduction in crystallinity and, thus, increasing fractions of amorphous GLB. This reveals that at least a partial amorphization of GLB occurs within the polymeric matrices during the process of hot-melt extrusion for these formulations.

**Figure 5:**
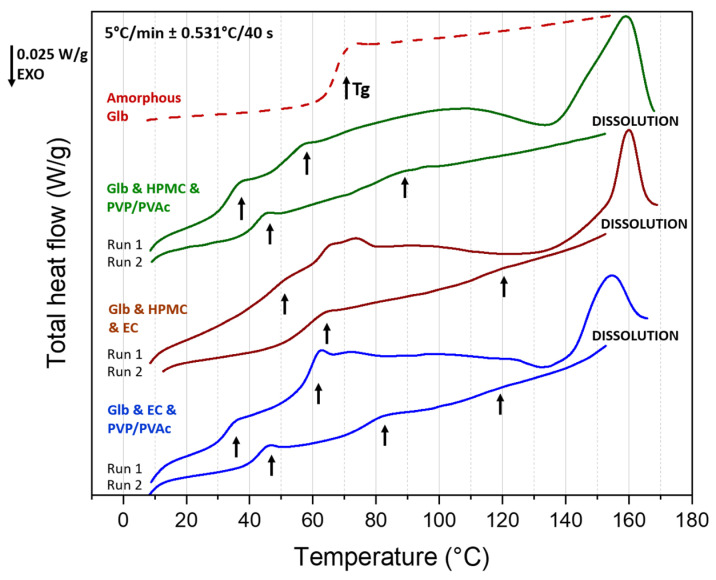
MDSC scans of ternary blend formulations containing 30% GLB: GLB and HPMC and PVP/PVAc represented in green, GLB and HPMC and EC in brown and GLB and EC and PVP/PVAc in blue. Thermogram of amorphous GLB is presented in red for comparison. The arrows highlight the glass transitions. Reversible and non-reversible heat flow curves are presented in the Supplementary Figures S2–S4.

Figure 5 shows the MDSC heating scans of all three investigated formulations after extrusion and the milled-induced amorphous glibenclamide (2nd heating scan-reversible heat flow, red curve) for comparison. The pure amorphous glibenclamide has a midpoint glass transition temperature of 67 ± 1 °C with a difference in heat capacity between the glassy and the liquid states (∆Cp) value of 0.44 ± 0.02 J/g °C. The determined glass transition temperature of milled glibenclamide is a bit lower than reported before by Patterson et al. [25] and Pei T. Mah et al. [21]. Since the UV-HPLC characterization of the milled sample excluded the presence of degradation products (that may have a plasticizing effect according to Patterson et al. [25]), we attributed this difference to the slower heating rate (5 °C/min instead of 10 °C/min).

For extruded materials, a first heating cycle was performed (run 1) up to 170 °C followed by cooling and subsequent heating (run 2) to help interpret the response once the sample has been dehydrated and the thermal history has been erased. Common features are observed in MDSC analyses of the blends. For the three extruded formulations, on the first heating, a large endothermic bump extending from room temperature to 135 °C can be seen on the total heat flow curves (cf. Figure 5). This indicates the overlapping of different thermal events. The analysis of the non-reversible heat flows (see Supplementary Figures S2–S4) and the disappearance of this wide endotherm on the second heating run indicate that it is mainly due to a loss of water, which agrees with TGA results. In the same temperature range, the analysis of the reversible heat flows (see Supplementary Figures S2–S4) reveals additional Cp-jumps, characteristic of glass transitions, which appear for some of them as peaks on the total heat flow curves (cf. Figure 5) because of superimposed overshoots of physical aging. At higher temperatures (135 °C–170 °C), an endothermic peak can be seen on the first scan for each blend. These peaks appeared at a lower temperature than the melting of GLB (Tm = 169 °C by MDSC), which indicates that they correspond to a dissolution endotherm. Indeed, as shown by XRD, crystalline GLB remained in the formulations after the HME process. When heated, GLB dissolves within the polymeric matrices starting at 135 °C for the formulations made of HPMC and PVP/PVAc and EC and PVP/PVAc and at 150 °C for the one made of HPMC and EC. It is interesting to note that the beginning of these dissolution processes roughly corresponds to the extrusion temperatures of the blends.

A more detailed analysis of run 1 in the temperature range where glass transitions are detected allowed to clearly observe a first Cp-jump at 36 ± 1 °C and a second at 57 ± 1 °C for GLB and HPMC and PVP/PVAc. Traces of a third one appear around 102 °C (see Supplementary Figure S2). On the second scan, only two Cp-jumps appeared at 45 ± 1 °C and 86 ± 1 °C. On the one hand, the first one matches the Tg of pure PVP/PVAc (determined at 44 ± 1 °C). For both runs, the first Cp-jump can thus be assigned to the glass transition of PVP/PVAc, plasticized by water, observable during the first heating. On the other hand, the second Cp-jump lies between the Tg of pure GLB (Tg = 67 ± 1 °C) and pure HPMC (Tg = 106 ± 1 °C). Since no recrystallization was observed upon cooling, it can be assigned to the glass transition of amorphous GLB and HPMC homogeneously mixed at the molecular scale. This suggests an affinity of GLB to diffuse within the polymer HPMC. Due to the plasticizing effect of water in the first heating run, it is difficult to conclude whether the two observed Cp-jumps at 57 °C and 102 °C are due to plasticized Tg of a small amorphous fraction of GLB and HPMC or plasticized Tg of GLB and HPMC blends of different compositions (one rich in GLB and the other in HPMC), respectively. In any case, it confirms that part of GLB has been amorphized during the HME process.

For GLB and HPMC and EC, during the first heating cycle, a significant Cp-jump was observed between 40 °C and 80 °C. In the second heating cycle, after dehydration and the dissolution of remaining crystalline GLB, the Cp-jump occurred within a narrower temperature range of 50 °C to 80 °C (see Supplementary Figure S3). The measured Tg is 62 ± 1 °C and can thus be attributed to the glass transition of GLB. This is five degrees lower than the Tg of pure GLB, which can be attributed to a plasticizing effect of the degradation products [25]. At higher temperatures, although not very pronounced, Cp-jumps corresponding to the glass transition of HPMC (around 103 °C) and EC (Tg = 122 °C) were detected in both runs. These results suggest that while part of GLB becomes amorphous during the HME process, this amorphous GLB is not mixed at the molecular level within HPMC or EC. The insufficient mixing, even after heating up to 170 °C in DSC, could be due to the high viscosity of the polymeric matrix.

For GLB and EC and PVP/PVAc, three Cp-jumps were detected in the first run: one at 35 ± 1 °C, assigned to the Tg of plasticized PVP/PVAc, which appeared at 44 °C in the second scan, similar to the blend with HPMC; one at 62 ± 1 °C, attributed to the glass transition of GLB, as in the blend GLB and HPMC and EC; and one at 118 ± 1 °C, assigned to the glass transition of EC. It should be noted that around 80 °C, the reversible flow signal is unclear, and the presence of a small Cp-jump cannot be excluded (see Supplementary Figure S4). This result indicates that during the HME process, a large portion of GLB became amorphous, an observation consistent with XRD analysis. However, this amorphous fraction did not blend with PVP/PVAc or EC at the extrusion temperature of 130 °C. During the second heating, the Tg of GLB disappeared, and instead, a Cp-jump was observed at higher temperatures (Tg = 81 ± 1 °C). Since only traces of the Tg of EC remain (at 116 ± 1 °C), this can be attributed to the glass transition of GLB and EC mixed at the molecular level, suggesting an affinity of GLB to diffuse into EC. The remaining Tg at 116 °C most likely corresponds to a blend of GLB and EC that is richer in EC, indicating a non-homogeneous GLB/EC blend after heating to 165 °C.

The MDSC analysis of the three formulations confirms that GLB undergoes partial amorphization during the HME process, even in smaller quantities in the case of the GLB and HPMC and PVP/PVAc blend. However, it also indicates that GLB is not blended at the molecular scale within the polymeric matrices. Upon the heating and dissolution of blends with HPMC and PVP/PVAc and EC and PVP/PVAc, the disappearance of the GLB glass transition and the appearance of a new one at higher temperatures suggest a greater affinity of GLB toward HPMC compared with EC, with no affinity toward PVP/PVAc.

### 3.4. Optical Appearance of Extruded Matrices via SEM Coupled with EDX Analysis

SEM imaging was conducted to examine the surface and cross-sectional morphology of the extrudates in the ternary blend containing glibenclamide, HPMC and PVP/PVAc.

**Figure 6:**
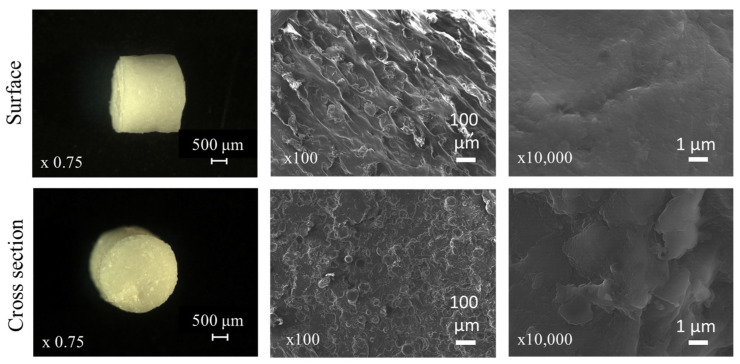
Optical macroscopy (**left**) and scanning electronic microscopy (**middle** and **right**) images of surfaces and cross sections of drug-loaded extrudates (based on HPMC and PVP/PVAc) before exposure to the release media.

The surface of the analyzed extrudates appears to be smooth, dense and homogeneous, with no crystalline drug particles observed at a 10,000-fold magnification. The striated appearance, visible in the middle row upper picture in Figure 6, results from the shearing of the molten mass against the die wall when exiting the extruder.

**Figure 7:**
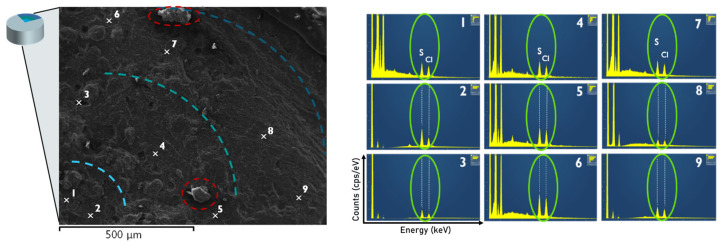
Scanning electronic microscopy image (**left**) coupled with energy dispersive X-ray (EDX) analysis performed in the different zones of the extrudate section (numbered from 1 to 9). It allows us to determine the distribution of GLB within the polymeric matrix of HPMC and PVP/PVAc by observing the presence of sulfur and chlorine atoms specific to GLB.

SEM imaging coupled with EDX analysis was performed to determine the distribution of glibenclamide within the polymeric matrix composed of HPMC and PVP/PVAc. Figure 7 shows the analysis, which examined the composition of different zones, starting from the interior and moving toward the exterior of the extrudate. The presence of sulfur and chlorine, elements specific to glibenclamide and absent in the polymers forming the matrix, was examined. Significant amounts of both elements, indicative of glibenclamide, were detected in all nine zones analyzed. These results suggest that the glibenclamide crystallites identified using X-ray diffraction were not located on the surface of the extrudates or clustered in specific areas but were homogeneously dispersed within the polymeric matrix.

It should be noted that the shapes circled in red in Figure 7 showed similarities to those observed by Pei T. Mah et al. [21] in partially amorphous GLB formulations. They discussed “nugget”-like crystals embedded within the porous amorphous matrix. These zones were investigated in greater detail; however, no presence of sulfur or chlorine was detected during the analysis. This leads to the conclusion that these “nugget”-like shaped forms observed on the SEM image are not glibenclamide crystallites.

### 3.5. Dissolution Kinetics and Optical Appearance of Ternary Blends Obtained by HME

**Figure 8:**
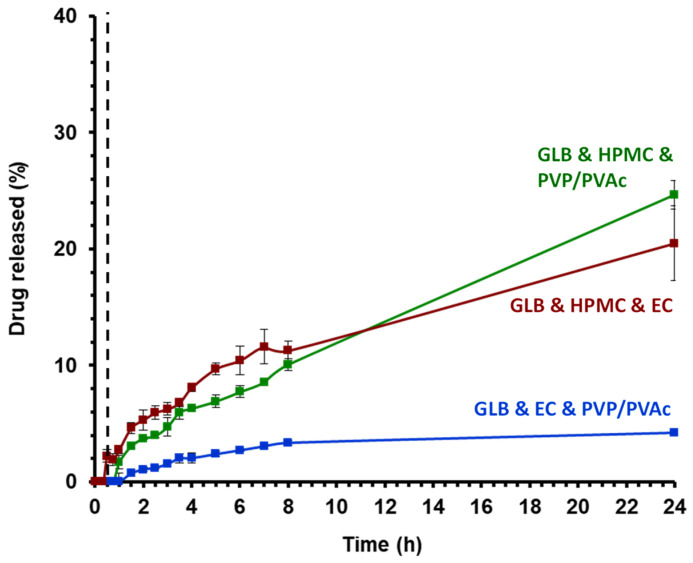
Drug release profiles of extruded ternary blends in a two-stage dissolution set-up (n = 3, USP II) for the first 30 min in HCl pH 1.2 (non-sink) and afterward in PB pH 6.8 at 37 °C under agitation (75 rpm) in sink conditions.

GLB is a weakly acidic drug with an estimated pKa of 5.3. Its acidic properties and pKa suggest a favorable absorption in basic media or at pH values above the pKa [39]. In order to observe the dissolution kinetics of GLB from the polymeric matrix obtained through HME, a two-stage dissolution protocol was implemented to perform a pH change comparable to the change observed from the gastric to the intestinal pH (cf. 2.8) [40]. The saturation concentration of GLB in both media (under the addition of 1% surfactant) was determined following the solubility tests protocol (cf. 2.8): in acidic conditions (pH 1.2), GLB can be considered practically insoluble in aqueous solutions with a saturation concentration of 6.1 ± 1.7 μg/mL. In contrast, in PB (pH 6.8), the saturation concentration was found to be almost three times higher, with a value of 17.25 ± 0.5 μg/mL. The results of GLB release rates from polymeric matrices are depicted in Figure 8. GLB release in acidic media is either undetectable or slow, with less than 3% released after 30 min in the case of GLB and HPMC and PVP/PVAc, consistent with the low saturation concentration measured in acidic conditions. Upon changing from HCl (pH 1.2) media to PB (pH 6.8), the release rate increased. Klumpp and Dressman explored the pH dependency of glibenclamide dissolution and confirmed that GLB dissolution behavior is primarily influenced by pH: higher pH values in the media lead to increased release rates [39]. The highest amounts of GLB released after 24 h was achieved with the HPMC and PVP/PVAc-based matrix (25%), followed by the EC and HPMC formulation (21%). The lowest release was observed with the EC and PVP/PVAc formulation (8%). GLB remained partially crystalline in all three formulations; however, XRD recordings indicate varying degrees of crystallinity (cf. Figure 4). Interestingly, the formulation that supposed the highest degrees of amorphous GLB, the EC and PVP/PVAc blend-based extrudate, exhibits the lowest release. Alonzo et al. have reported on the phenomenon where the dissolution advantage of amorphous solids is negated by crystallization when in contact with the dissolution medium [41]. However, the authors hypothesize that this is not the case in the present study and that the release rate is more likely related to the particular characteristics of the different polymers employed to formulate the polymeric matrices.

**Figure 9:**
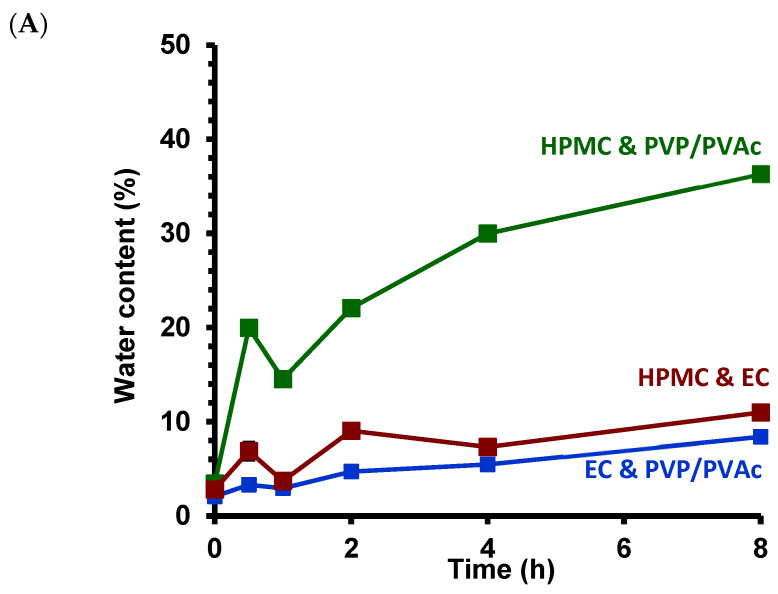

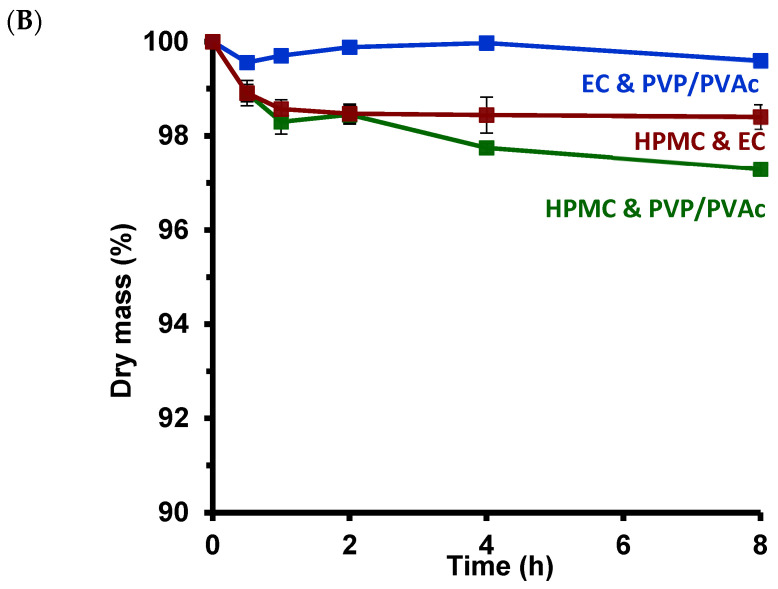
Water uptake (**A**) and dry mass loss (**B**) of the extrudates based on the three blends investigated (as indicated).

Indeed, the mechanism and kinetics of drug release are determined by the rate of swelling (relaxation) of hydrated polymer, as well as the diffusion and erosion of the polymeric matrices [42]. GLB released from the polymeric matrices EC and HPMC and HPMC and PVP/PVAc showed similar release behavior. In studies of water uptake and dry mass loss (Figure 9), the HPMC and PVP/PVAc blend demonstrated the highest swelling properties (up to 36.3%) and the most important polymer erosion (2.7% loss in mass) among the three formulations investigated. In contrast, PVP/PVAc and EC show the lowest water uptake (8.4%) and dry mass loss (0.4%).

**Figure 10:**
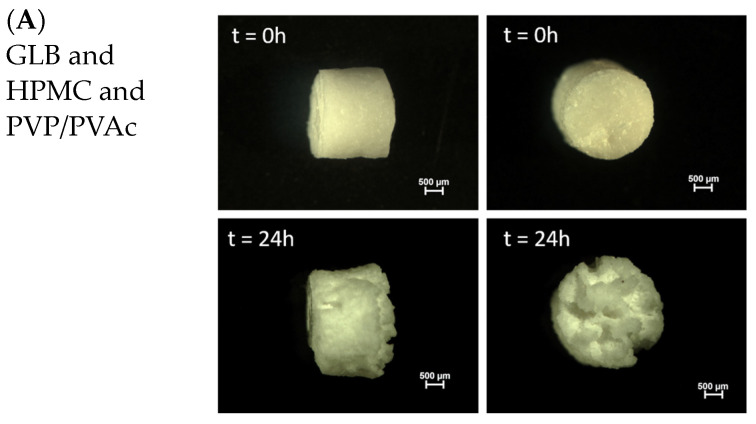

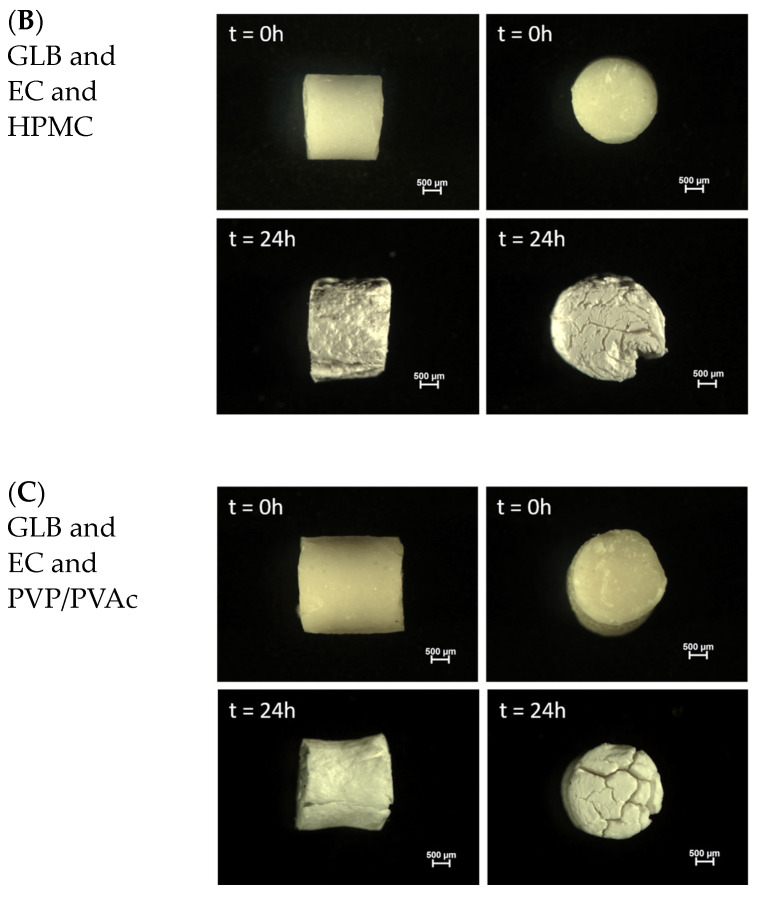
Optical macroscopy pictures of extrudates based on (**A**) HPMC and PVP/PVAc, (**B**) EC and HPMC and (**C**) EC and PVP/PVAc before and after exposure to release medium.

Regarding the mass transport mechanisms to be associated with PVP/PVAc, Siepmann et al. reported drug diffusion with constant diffusivity in Kollidon SR-based matrix tablets [43]. Aita et al. formulated a sustained-release formulation where the drug was encapsulated within a polymeric matrix composed of Kollidon SR and HPMC. They reported that the dissolution behavior of their printed tablets is controlled by matrix swelling followed by the release of the API by diffusion. Over time, contact with the medium leads to erosion of the matrix [44]. Figure 10A shows optical macroscopy images of GLB and HPMC and PVP/PVAc extrudates before and after exposure to the release media during the two-step dissolution test. Initially, the surface and cross-section appear smooth without visible cracks or artifacts. However, after 24 h in the dissolution media, the matrices exhibited significant visual changes. The extrudate developed a “coral”-like shape, suggesting that water penetrates the polymer upon contact with the media, causing the extrudate to gelatinize and swell, particularly due to the presence of HPMC in the formulation. This swelling creates pathways through which the API can diffuse. The formation of cracks and the “coral”-like shape could serve as channels through which the aqueous media can get into contact with embedded API, facilitating its release.

De Brabander et al. studied the impact of an extruded HPMC and EC matrix on drug release [45]. They reported that drug release was not predominantly controlled by erosion effects but rather follows an anisotropic swelling behavior induced by stresses during the HME process. HPMC exhibits greater swelling capacity compared with lipophilic ethyl cellulose (EC), allowing HPMC to enhance water penetration into the polymeric matrix [45]. Furthermore, Crowley et al. studied the influence of direct compression and HME on the drug release mechanism of EC matrices. HME formulations showed a delayed release attributed to smaller pore sizes, which were less porous and twisted within the matrices [46]. Figure 10B depicts the optical appearance of the HPMC and EC blend-based extrudates, exhibiting thinner cracks on the surface. However, it is important to consider that these cracks can be the result of sample drying after swelling. Figure 10C shows macroscopic images of the blend of EC and PVP/PVAc polymers, underlining the hypothesized release mechanisms discussed previously. The images clearly show deep cracks within the polymeric matrix. In conclusion, the hot-melt extrusion process creates challenges for medium penetration due to smaller porosity in general (compared to compressed matrices, for example), and the presence of HPMC within the matrix formulations improves drug release kinetics.

## 4. Conclusions

The present study illustrates the influence of hot-melt extrusion on the physical and chemical properties of the heat-labile drug glibenclamide. The applied process parameters did not result in complete drug recovery. Therefore, the study of degradation was conducted to enhance understanding and optimize future processes. This work facilitates the identification of impurity A and the quantification of the extent of degradation following the HME process. Besides the effects of heat and shear, the small amount of water absorbed by the hygroscopic polymers could lead to hydrolysis of the drug. In addition, physical characterization of the extrudates pointed out the presence of GLB crystallites alongside the amorphous form, indicating that only partial amorphization was achieved. DSC studies revealed that the remaining crystalline glibenclamide dissolves within the polymers composing the matrices and that the beginning of this dissolution process coincides with the temperatures at which extrusion became processable for the three blends investigated. Moreover, drug release studies revealed a primary dependency on pH and the specific characteristics of the polymeric matrix rather than the physical state of the drug. The HPMC and PVP/PVAc matrix exhibited the highest water uptake and highest drug release among the formulations investigated. In conclusion, this study provides a better understanding of the comportment of glibenclamide during the HME process and its dissolution performance, which could be applicable to other sulfonylurea-based antidiabetic drugs.

## Figures and Tables

**Table 1:** HME processing conditions and drug recovery within the extrudates.

Ternary Blend Composition	Feeding Rate [g/min]	Screw Speed [rpm]	Torque [%]	Extrudate Output [g/min]	T Zone 2 [°C]	T Zone 3 [°C]	T Zone 4–6 [°C]	T Zone 7 [°C]	T Zone 8 [°C]	T Die [°C]	Drug Recovery [%]
GLB and HPMC and PVP/PVAc	1.71	160	60	1.67	60	100	110	115	120	130	75.0 ± 6.7
GLB and EC and PVP/PVAc	1.92	200	40	1.85	60	100	110	110	125	130	74.9 ± 8.2
GLB and HPMC and EC	1.06	45	75	0.81	110	130	150	150	150	155	72.4 ± 10.8

## Data Availability

Data are contained within the article or Supplementary Materials.

## Supplementary Materials

The following supporting information can be downloaded at https://www.mdpi.com/article/10.3390/pharmaceutics16081071/s1, Figure S1: Screw configuration used for all three extrusion runs; Figure S2: MDSC scans of ternary blend formulation containing 30% GLB: GLB and HPMC and PVP/PVAc represented in green. Thermogram of amorphous GLB is presented in red for comparison. Different heat flows are represented on the graph: total heat flow (―), reversible heat flow (---) and non-reversible heat flow (-ꞏ-); Figure S3: MDSC scans of ternary blend formulation containing 30% GLB: GLB and HPMC and EC represented in brown. Different heat flows are represented on the graph: total heat flow (―), reversible heat flow (---) and non-reversible heat flow (-ꞏ-); Figure S4: MDSC scans of ternary blend formulation containing 30% GLB: GLB and EC and PVP/PVAc represented in blue. Different heat flows are represented on the graph: total heat flow (―), reversible heat flow (---) and non-reversible heat flow (-ꞏ-).

## References

[1] Choiri S., Sulaiman T.N.S., Rohman A. (2019). Characterization of Eudragit types and Kollidon SR inter-polymer complexes and their effects on the drug release. J. Appl. Pharm. Sci..

[2] Borde S., Paul S.K., Chauhan H. (2021). Ternary solid dispersions: Classification and formulation considerations. Drug Dev. Ind. Pharm..

[3] Crowley M.M., Zhang F., Repka M.A., Thumma S., Upadhye S.B., Battu S.K., McGinity J.W., Martin C. (2007). Pharmaceutical Applications of Hot-Melt Extrusion: Part I. Drug Dev. Ind. Pharm..

[4] Thakkar R., Thakkar R., Pillai A., Ashour E.A., Repka M.A. (2020). Systematic screening of pharmaceutical polymers for hot melt extrusion processing: A comprehensive review. Int. J. Pharm..

[5] Censi R., Gigliobianco M., Casadidio C., Di Martino P. (2018). Hot Melt Extrusion: Highlighting Physicochemical Factors to Be Investigated While Designing and Optimizing a Hot Melt Extrusion Process. Pharmaceutics.

[6] Stanković M., Frijlink H.W., Hinrichs W.L.J. (2015). Polymeric formulations for drug release prepared by hot melt extrusion: Application and characterization. Drug Discov. Today.

[7] Van Den Mooter G. (2012). The use of amorphous solid dispersions: A formulation strategy to overcome poor solubility and dissolution rate. Drug Discov. Today Technol..

[8] He Y., Ho C. (2015). Amorphous Solid Dispersions: Utilization and Challenges in Drug Discovery and Development. J. Pharm. Sci..

[9] Kolter K., Karl M., Gryczke A. (2012). Hot-Melt Extrusion with BASF Pharma Polymers.

[10] Matić J., Alva C., Witschnigg A., Eder S., Reusch K., Paudel A., Khinast J. (2020). Towards predicting the product quality in hot-melt extrusion: Small scale extrusion. Int. J. Pharm..

[11] Prasad E., Islam M.T., Goodwin D.J., Megarry A.J., Halbert G.W., Florence A.J., Robertson J. (2019). Development of a hot-melt extrusion (HME) process to produce drug loaded Affinisol^TM^ 15LV filaments for fused filament fabrication (FFF) 3D printing. Addit. Manuf..

[12] Kukkonen J., Ervasti T., Laitinen R. (2022). Production and characterization of glibenclamide incorporated PLA filaments for 3D printing by fused deposition modeling. J. Drug Deliv. Sci. Technol..

[13] Alshafiee M., Aljammal M.K., Markl D., Ward A., Walton K., Blunt L., Korde S., Pagire S.K., Kelly A., Paradkar A. (2019). Hot-melt extrusion process impact on polymer choice of glyburide solid dispersions: The effect of wettability and dissolution. Int. J. Pharm..

[14] Liu J., Cao F., Zhang C., Ping Q. (2013). Use of polymer combinations in the preparation of solid dispersions of a thermally unstable drug by hot-melt extrusion. Acta Pharm. Sin. B.

[15] Huang S., O’Donnell K.P., Delpon De Vaux S.M., O’Brien J., Stutzman J., Williams R.O. (2017). Processing thermally labile drugs by hot-melt extrusion: The lesson with gliclazide. Eur. J. Pharm. Biopharm..

[16] Baronsky-Probst J., Möltgen C.-V., Kessler W., Kessler R.W. (2016). Process design and control of a twin screw hot melt extrusion for continuous pharmaceutical tamper-resistant tablet production. Eur. J. Pharm. Sci..

[17] Bansal G., Singh M., Jindal K.C., Singh S. (2008). Ultraviolet-Photodiode Array and High-Performance Liquid Chromatographic/Mass Spectrometric Studies on Forced Degradation Behavior of Glibenclamide and Development of a Validated Stability-Indicating Method. J. AOAC Int..

[18] Iyer J., Brunsteiner M., Modhave D., Paudel A. (2023). Role of Crystal Disorder and Mechanoactivation in Solid-State Stability of Pharmaceuticals. J. Pharm. Sci..

[19] DiNunzio J.C., Brough C., Hughey J.R., Miller D.A., Williams R.O., McGinity J.W. (2010). Fusion production of solid dispersions containing a heat-sensitive active ingredient by hot melt extrusion and Kinetisol^®^ dispersing. Eur. J. Pharm. Biopharm..

[20] Hughey J.R., DiNunzio J.C., Bennett R.C., Brough C., Miller D.A., Ma H., Williams R.O., McGinity J.W. (2010). Dissolution Enhancement of a Drug Exhibiting Thermal and Acidic Decomposition Characteristics by Fusion Processing: A Comparative Study of Hot Melt Extrusion and KinetiSol^®^ Dispersing. AAPS PharmSciTech.

[21] Mah P.T., Laaksonen T., Rades T., Aaltonen J., Peltonen L., Strachan C.J. (2014). Unravelling the Relationship between Degree of Disorder and the Dissolution Behavior of Milled Glibenclamide. Mol. Pharm..

[22] Wojnarowska Z., Grzybowska K., Adrjanowicz K., Kaminski K., Paluch M., Hawelek L., Wrzalik R., Dulski M., Sawicki W., Mazgalski J. (2010). Study of the Amorphous Glibenclamide Drug: Analysis of the Molecular Dynamics of Quenched and Cryomilled Material. Mol. Pharm..

[23] Laitinen R., Löbmann K., Grohganz H., Strachan C., Rades T. (2014). Amino Acids as Co-amorphous Excipients for Simvastatin and Glibenclamide: Physical Properties and Stability. Mol. Pharm..

[24] Hassan M.A., Najib N.M., Suleiman M.S. (1991). Characterization of glibenclamide glassy state. Int. J. Pharm..

[25] Patterson J.E., James M.B., Forster A.H., Lancaster R.W., Butler J.M., Rades T. (2005). The Influence of Thermal and Mechanical Preparative Techniques on the Amorphous State of Four Poorly Soluble Compounds. J. Pharm. Sci..

[26] Rehder S., Sakmann A., Rades T., Leopold C.S. (2012). Thermal degradation of amorphous glibenclamide. Eur. J. Pharm. Biopharm..

[27] Yuce C. Twin-Screw Extrusion Applications in the Pharmaceutical Industry. Proceedings of the Master 2 Pharmacie Galénique Industrielle Program Formation.

[28] Bassand C., Benabed L., Freitag J., Verin J., Siepmann F., Siepmann J. (2022). How bulk fluid renewal can affect in vitro drug release from PLGA implants: Importance of the experimental set-up. Int. J. Pharm. X.

[29] Gupta S.S., Solanki N., Serajuddin A.T.M. (2016). Investigation of Thermal and Viscoelastic Properties of Polymers Relevant to Hot Melt Extrusion, IV: Affinisol^TM^ HPMC HME Polymers. AAPS PharmSciTech.

[30] Huang S., O’Donnell K.P., Keen J.M., Rickard M.A., McGinity J.W., Williams R.O. (2016). A New Extrudable Form of Hypromellose: AFFINISOL^TM^ HPMC HME. AAPS PharmSciTech.

[31] Prodduturi S., Urman K.L., Otaigbe J.U., Repka M.A. (2007). Stabilization of hot-melt extrusion formulations containing solid solutions using polymer blends. AAPS PharmSciTech.

[32] Martin C., Repka M.A., Langley N., DiNunzio J. (2013). Twin Screw Extrusion for Pharmaceutical Processes. Melt Extrusion.

[33] Hanada M., Jermain S.V., Thompson S.A., Furuta H., Fukuda M., Williams R.O. (2021). Ternary Amorphous Solid Dispersions Containing a High-Viscosity Polymer and Mesoporous Silica Enhance Dissolution Performance. Mol. Pharm..

[34] Thompson S.A., Williams R.O. (2021). Specific mechanical energy—An essential parameter in the processing of amorphous solid dispersions. Adv. Drug Deliv. Rev..

[35] Radjenović J., Pérez S., Petrović M., Barceló D. (2008). Identification and structural characterization of biodegradation products of atenolol and glibenclamide by liquid chromatography coupled to hybrid quadrupole time-of-flight and quadrupole ion trap mass spectrometry. J. Chromatogr. A.

[36] Haynes W.M. (2016). CRC Handbook of Chemistry and Physics.

[37] Budavari S., O’Neil M.J., Smith A., Heckelman P.E., Obenchain J.R., Gallipeau J.A.R., D’Arecea M.A. (1996). The Merck Index: An Encyclopedia of Chemicals, Drugs, and Biologicals.

[38] Suresh K., Khandavilli U.B.R., Gunnam A., Nangia A. (2017). Polymorphism, isostructurality and physicochemical properties of glibenclamide salts. CrystEngComm.

[39] Klumpp L., Dressman J. (2020). Physiologically based pharmacokinetic model outputs depend on dissolution data and their input: Case examples glibenclamide and dipyridamole. Eur. J. Pharm. Sci..

[40] Mann J., Dressman J., Rosenblatt K., Ashworth L., Muenster U., Frank F., Hutchins P., Williams J., Klumpp L., Wielockx K. (2017). Validation of Dissolution Testing with Biorelevant Media: An OrBiTo Study. Mol. Pharm..

[41] Alonzo D.E., Zhang G.G.Z., Zhou D., Gao Y., Taylor L.S. (2010). Understanding the Behavior of Amorphous Pharmaceutical Systems during Dissolution. Pharm. Res..

[42] Langer R., Peppas N. (1983). Chemical and Physical Structure of Polymers as Carriers for Controlled Release of Bioactive Agents: A Review. J. Macromol. Sci..

[43] Siepmann F., Eckart K., Maschke A., Kolter K., Siepmann J. (2010). Modeling drug release from PVAc/PVP matrix tablets. J. Control. Release.

[44] Aita I.E., Breitkreutz J., Quodbach J. (2020). Investigation of semi-solid formulations for 3D printing of drugs after prolonged storage to mimic real-life applications. Eur. J. Pharm. Sci..

[45] De Brabander C., Vervaet C., Remon J.P. (2003). Development and evaluation of sustained release mini-matrices prepared via hot melt extrusion. J. Control. Release.

[46] Crowley M.M., Schroeder B., Fredersdorf A., Obara S., Talarico M., Kucera S., McGinity J.W. (2004). Physicochemical properties and mechanism of drug release from ethyl cellulose matrix tablets prepared by direct compression and hot-melt extrusion. Int. J. Pharm..

