# Supplementary material for: Impact of Hot-Melt Extrusion on Glibenclamide’s Physical and Chemical States and Dissolution Behavior: Case Studies with Three Polymer Blend Matrices
Source: Pharmaceutics. 2024 Aug 15;16(8):1071. doi: 10.3390/pharmaceutics16081071 (PMC11360095; doi:10.3390/pharmaceutics16081071)
Supplement: Supplementary file 1 [file pharmaceutics-16-01071-s001.zip › pharmaceutics-3134635-supplementary.pdf]

pharmaceutics

MDPI

Supplementary

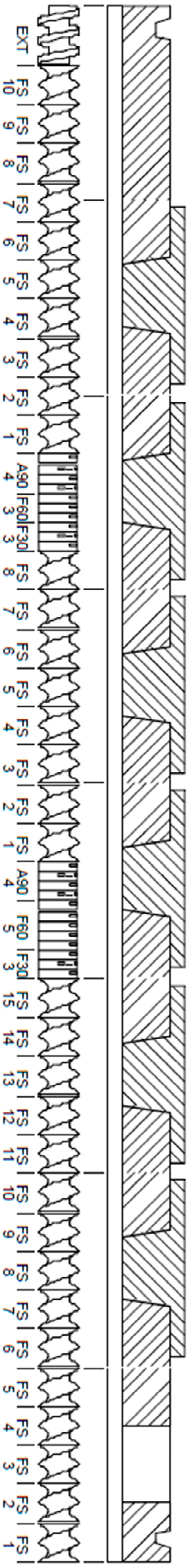

Figure S1. Screw configuration used for all extrusion experiments.

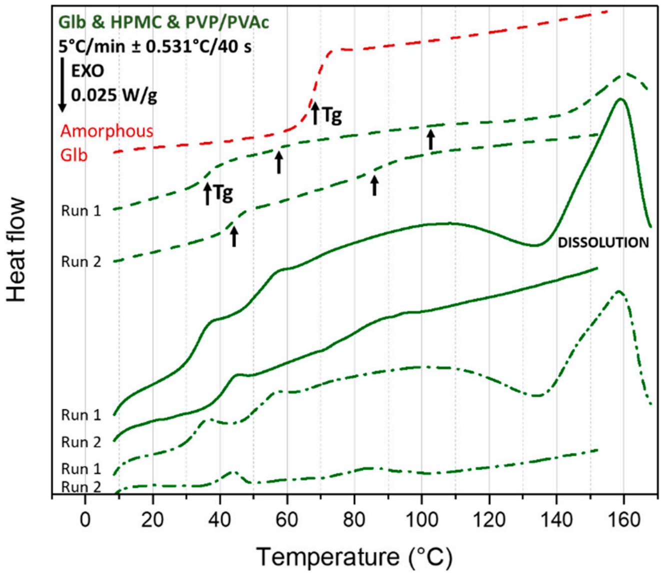

Figure S2. MDSC scans of ternary blend formulation containing 30% GLB: GLB & HPMC & PVP/PVAc represented in green. Thermogram of amorphous GLB is presented in red for comparison. Different heat flows are represented on the graph: total heat flow (—), reversible heat flow (---) and non-reversible heat flow (-··-).

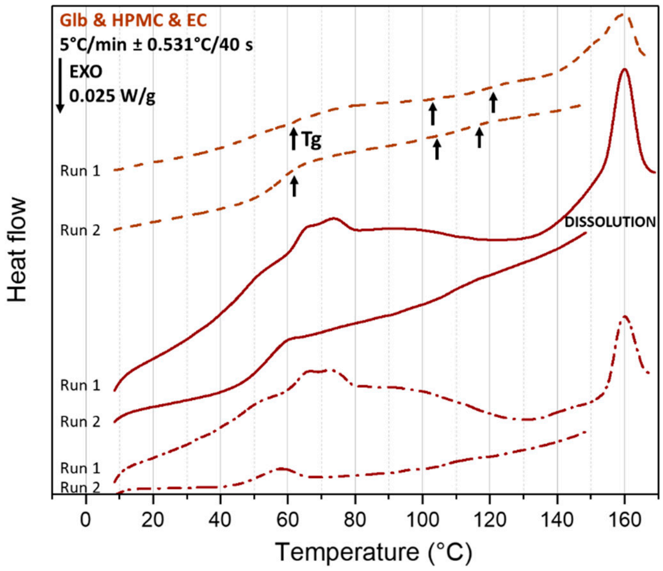

Figure S3. MDSC scans of ternary blend formulation containing 30% GLB: GLB & HPMC & EC represented in brown. Different heat flows are represented on the graph: total heat flow (—), reversible heat flow (---) and non-reversible heat flow (-··-).

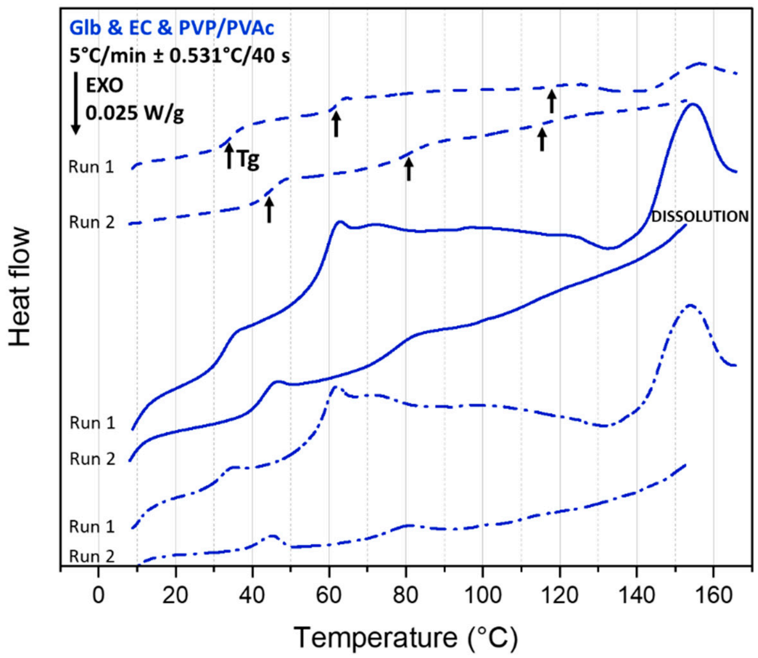

Figure S4. MDSC scans of ternary blend formulation containing 30% GLB: GLB & EC & PVP/PVAc represented in blue. Different heat flows are represented on the graph: total heat flow (—), reversible heat flow (---) and non-reversible heat flow (-·-).
